# Deep Learning Based Fast Screening Approach on Ultrasound Images for Thyroid Nodules Diagnosis

**DOI:** 10.3390/diagnostics11122209

**Published:** 2021-11-26

**Authors:** Hafiz Abbad Ur Rehman, Chyi-Yeu Lin, Shun-Feng Su

**Affiliations:** 1 Department of Mechanical Engineering, National Taiwan University of Science and Technology, Taipei 106, Taiwan; jerrylin@mail.ntust.edu.tw; 2 Department of Electrical Engineering, National Taiwan University of Science and Technology, Taipei 106, Taiwan; sfsu@mail.ntust.edu.tw

**Keywords:** deep learning, thyroid nodule, healthcare, medical diagnosis

## Abstract

Thyroid nodules are widespread in the United States and the rest of the world, with a prevalence ranging from 19 to 68%. The problem with nodules is whether they are malignant or benign. Ultrasonography is currently recommended as the initial modality for evaluating thyroid nodules. However, obtaining a good diagnosis from ultrasound imaging depends entirely on the radiologists levels of experience and other circumstances. There is a tremendous demand for automated and more reliable methods to screen ultrasound images more efficiently. This research proposes an efficient and quick detection deep learning approach for thyroid nodules. An open and publicly available dataset, Thyroid Digital Image Database (TDID), is used to determine the robustness of the suggested method. Each image is formatted into a pyramid tile-based data structure, which the proposed VGG-16 model evaluates to provide segmentation results for nodular detection. The proposed method adopts a top-down approach to hierarchically integrate high- and low-level features to distinguish nodules of varied sizes by employing fuse features effectively. The results demonstrated that the proposed method outperformed the U-Net model, achieving an accuracy of 99%, and was two times faster than the competitive model.

## 1. Introduction

The thyroid gland is a butterfly-shaped endocrine gland in the lower front of the neck [1]. This endocrine gland produces thyroid hormones, which are then released into the bloodstream and help maintain the human body’s metabolism [2]. Thyroid cancer is increasing worldwide, while the fatality rate remains steady [3]. Thyroid nodules are relatively common with a prevalence of 19–68% in the general population and are usually discovered incidentally in the first routine neck imaging scan [3,4]. In 2019, around 52,070 people in the United States were diagnosed with thyroid cancer; among them, females are more prevalent than males [5].

There have been numerous initiatives in the last ten years to reduce the prevalence of thyroid cancer [6,7]. Ultrasound imaging is the most commonly used technique in thyroid radiology for thyroid nodule assessment due to its non-invasive nature and cost-effectiveness [8]. The most significant factor in a successful surgery outcome is accurate thyroid nodule diagnosis. Besides conventional methods in clinical diagnostics, computer-aided diagnosis (CAD) systems have become more common [9]. Digitalizing disease detection aims to achieve high accuracy for the diagnosis process and reduce patient cost and time. Various Machine Learning (ML) methods have been utilized for the advancement of the thyroid ultrasound CAD system. Deep Learning (DL), a subdomain of ML, has rapidly grown in medical imaging analysis and computer vision and is frequently seen as a viable alternative for evaluating ultrasound images [10].

Previous researchers have presented various approaches to detect nodules in ultrasound images. The authors of [11] presented a CAD system that applied a histogram analysis and segmentation-based fractal texture analysis algorithm for measuring the direction-independent features in ultrasound images to detect thyroid nodules. To distinguish between malignant and benign thyroid nodules, a support vector machine (SVM) and a random forest classifier were employed to extract characteristics. The authors also used segmentation techniques to improve nodules’ classification for more accurate diagnoses. Researchers Nougroho et al. [12] developed a CAD system to diagnose thyroid cancer. The primary purpose was to facilitate the radiologists in analyzing important characteristics of ultrasound images using a digital image processing method. Their proposed method had four stages: image enhancement, segmentation, feature extraction, and classifying each characteristic using multilayer perceptron (MLP) and SVM, and determining whether the tumor is benign or malignant. Song et al., in [13], introduced the InceptionV3-based approach for detecting thyroid nodules. The primary purpose of their research was to assist medical experts in identifying benign nodules and avoiding unnecessary Fine Needle Aspiration (FNA). They trained their algorithm on a shallow cropped nodule dataset created with the help of a physician. The performance of their experiment showed that their model might assist radiologists in recognizing malignant nodules with promising results. Authors Ko, S.Y. et al. [14] presented the convolutional neural network (CNN) model for thyroid cancer malignancy detection and compared the model output with the radiologists’ diagnostic performance. Two pre-trained models, i.e., “imagenet-vgg-verydeep16” and “imagenet-vgg-f” were used. A radiologist extracted the region of interest (ROI) from each ultrasound image to train the CNN with their local data. Results showed that both CNN performed similarly to expert radiologists’ images in differentiating thyroid cancer. The authors of [15] present a novel CAD system for categorizing and detecting thyroid ultrasound images driven by task-specific knowledge. The approach they proposed is divided into two parts. First, a multi-scale region-based detection network was built to learn pyramidal features for recognizing nodules at different scales. The following step was to create a multi-branch classification network with multi-view diagnosis-oriented features. Each network branch improved on a specific set of features that radiologists commonly employ. The authors claimed that their proposed CAD system outperformed 8% of expert radiologists’ findings. Vasile, M.C. et al. [16] published another study on diagnosing and classifying four different forms of thyroid nodules. They used an ensemble approach that combined two deep learning models for this purpose. Results showed that the proposed ensemble CNN–VGG technique outperformed the 5-CNN and VGG-19 models, achieving an overall accuracy of 97.35%. For an automatic diagnosis of thyroid nodules, a multitask cascade deep learning model (MCDLM) was presented by Yang, W. et al. [17], which integrated radiologists’ diverse domain knowledge (DK) and leveraged multimodal ultrasound images. The authors used the U-Net model and the dual-path semi-supervised conditional generative adversarial network (DScGAN) model for the precise segmentation results to generate high-quality images for discriminative purposes. After that, DScGAN generated images trained for a supervised support vector machine (S3VM) for thyroid nodule classification. Results showed that MCDLM achieved 90.01% classification accuracy. Another author, Abdolali, F. et al. [18], proposed an approach capable of detecting a variety of thyroid nodules. The proposed multitask model, Mask R-CNN, used regularization with a loss function and prioritized detection over-segmentation. Their suggested model outperformed Faster R-CNN’s and the traditional Mask R-CNN’s results.

Automatic precise detection of thyroid nodules is a crucial but challenging step for several reasons, i.e., blurry appearance, vague margin, irregular shape, and difficulty distinguishing between healthy tissues and nodule region. This research presented an automated technique for detecting and segmenting thyroid nodules using ultrasound images. The suggested approach uses a DL model with a fully convolutional neural network and a VGG-16 backbone to improve detection accuracy, and utilizing customized VGG-16 model results in achieving higher accuracy with a comparably simple model. We tested our model using several quantitative measures using a free and publicly available thyroid nodule dataset. Ground truth analysis was used to validate the thyroid nodule segmentation results.

The main contributions of our research consist of:

- The proposed approach can precisely segment the thyroid nodule from ultrasound images despite blurring and noise effect fluctuations in input images.
- The dataset employed for this study has diverse characteristics, consisting of 400 thyroid ultrasound images from five separate diagnosis stages, which are indicated by Thyroid Imaging Reporting and Data System (TIRADS)-1 to TIRADS-5.
- The annotation is created for the ground truth masks because the current dataset partially lacks labeling work.
- To validate the effectiveness of the proposed method, a statistical analysis of the proposed model in comparison with the U-Net model was provided.

The remaining sections are described as follows. Section 2 explains the methodology of the proposed convolutional neural network and its architecture. Subsequently, in Section 3, results with quantitative analysis of the proposed method and its comparison with other studies are presented. Finally, Section 4 concludes the paper with a future perspective.

## 2. Methodology

**Figure 1:**
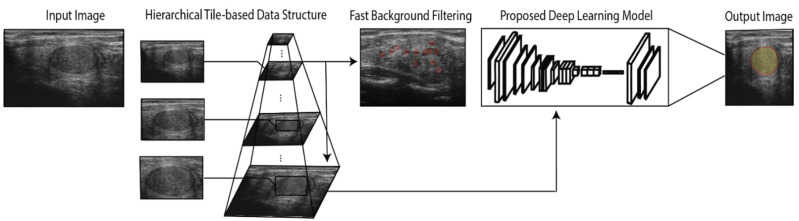
Schematic block diagram of the proposed methodology.

**Figure 2:**
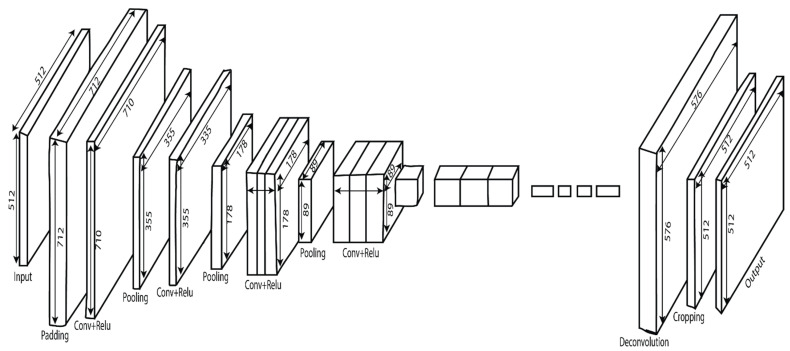
Proposed backbone VGG-16 architecture.

This Section presents the proposed method employed for thyroid nodule detection. The proposed convolutional neural network (CNN) used VGG-16 architecture as the backbone to process the ultrasound images [19]. Figure 1 depicts the methodology workflow. Each nodule image is first converted into a hierarchical tile-based data structure and processed to access the results of the nodule segmentation via the proposed CNN. Figure 2 shows the detailed architecture of the applied VGG-16 model. Furthermore, the effectiveness of the proposed approach was compared to that of another popular modern U-Net model [20].

### 2.1. Dataset

Collecting a significant amount of thyroid nodules-based ultrasound images is challenging due to time constraints and patient cooperation. Therefore, we chose to use a publicly available thyroid nodules images dataset. The Thyroid Digital Image Database (TDID) is an open and public dataset of Universidad Nacional de Colombia [21]. The TDID dataset, consisting of 400 ultrasonography thyroid images from 298 patients, was published in 2015. For each patient, one or more ultrasound images of the thyroid were obtained. The image size is 560 × 360 pixels, and it includes a detailed explanation and diagnostic description of the suspected thyroid lesions written by radiologists. The Thyroid Imaging Reporting and Data System (TI-RADS) of the American College of Radiology [22] scores were awarded to each image to predict the risk of thyroid nodule malignancy based on ultrasound parameters. TI-RADS level is a benchmark for evaluating the stage of the thyroid nodules, which aids in placing them in one of the five different stages. TIRADS-1 indicates the benign class, whereas TIRADS-5 indicates a significant risk of thyroid cancer.

### 2.2. Annotation

**Figure 3:**
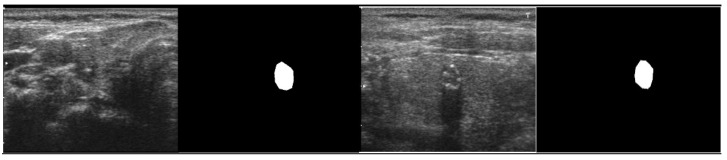
Original image and its corresponding GT masks.

The ground truth (GT) mask associated with each thyroid ultrasound picture is required to differentiate the nodule part for the training procedure. The labelme [23] annotates the thyroid images before creating a polygon mask for each image. Figure 3 shows an example of the original image and its GT counterpart. The interpretations of ultrasound images by labelme software are saved as JSON files, comprised of polygon points for the nodule region attributed to 0 or 1. The pixels inside the enclosing polygon associated with the nodule region have a value of 1, while the remainder are considered background having a value of 0.

### 2.3. Training Methodology

Table 1 demonstrates the distribution of the dataset for training and testing purposes in the proposed method. The proposed VGG-16 model employs a stochastic gradient descent (SGD) optimizer and a cross-entropy loss function for training. In contrast, the benchmark U-Net model employed an Adadelta optimizer and a cross-entropy loss function and used the Keras [24] framework for implementation. The learning rate adjustment throughout the training phase ensures maximum training accuracy and less training loss. The ideal learning rate would result in a rapid decrease in training loss until it reaches the minimum level. Table 2 lists the details of the proposed and benchmark models’ training parameters, such as learning rate, drop rate, weight decay, and optimizer.

### 2.4. Proposed Convolutional Neural Network (CNN) Architecture

The extraction of the core features can be carried out automatically using the convolutional neural network filters (CNN). For better training results, the implementation and adjustment of weights are essential. The proposed model architecture and its detailed configuration about filter size, padding, stride, and pooling are explained in Table 3. The input image given to CNN has 712 × 712 × 1 size after applying the padding operation. Five convolutional layers process the input images with Relu functions and pooling layers. The first two layers of CNN contain a sequence of two convolutional layers with 64 and 128 filters, respectively, with 3 × 3 kernel size and 1 × 1 stride size. However, the last three convolutional layers for three convolution sequences contain 3 × 3 kernel size and 1 × 1 stride size and use 256 filters for the third layer and 512 filters for the fourth and fifth layers. Before passing through the sequence of two drop-out layers, a feature map of 23 × 23 × 512 is obtained. The size of the output feature map can be calculated by using the following formula

(1) $$q_{h},q_{w},q_{r}=\left(\;\lfloor \frac{n_{h}+2e-k\;}{s}+1\rfloor ,\lfloor \frac{n_{w}+2e-k\;}{s}+1\rfloor ,\;n_{k}\right)$$

$n_{h}$ and $n_{w}$ represent the height and width of the input image size, respectively, whereas k denotes the kernel size, while $e,s,\;\mathrm{and}\;n_{k}$ indicate the padding size, stride size, and the number of filters used, respectively. $q_{h},q_{w},\mathrm{and}\;q_{r}$ represent the output height, width, and channel number after each convolutional layer, respectively. Pool-1 to Pool-5 layers performed the max-pooling function (kernel size 2 × 2, and stride 2 × 2) to reduce the feature map size. The output size of the image after each pooling layer can be formulated as follows

(2) $$d_{h},d_{w},d_{r}=\left(\;\lfloor \frac{n_{h}+2e-k\;}{s}+1\rfloor ,\lfloor \frac{n_{w}+2e-k\;}{s}+1\rfloor ,\;n_{c}\right)$$

where $n_{c}$ is the number of channels of the input. $d_{h},\;d_{w},\;\mathrm{and}\;d_{r}$ are the output height, width, and number of channel after pooling layers, respectively.

The two drop-out layers consisted of 4096 filters, kernel size of 7 × 7 and stride size 1 × 1. Following the drop-out layers, a convolution layer was used to decrease the number of output channels with 1 × 1 kernel size and 1 × 1 stride size. To resize the feature maps into the same padding images and predict each pixel while keeping the spatial information intact in both the original images and the upsampled feature maps, a deconvolutional layer with the configuration of 64 × 64 kernel size and stride size 1 × 1 is used. A cropping operation was performed after the deconvolution layer to fit the input size.

## 3. Results

This Section describes the testing and validation performance of the proposed CNN model based on accuracy, precision, and other standard classification metrics.

### 3.1. Evaluation Metrics

The segmentation results are quantitatively evaluated using parameters such as accuracy (Acc), intersection-over-union (IoU), precision, recall, and dice score (DSC). The explanation of these parameters is as follows

(3) $$Accuracy\;\left(Acc\right)=\frac{TP+TN}{TP+TN+FP+FN}\;$$

(4) $$Sensitivity\;or\;Recall\;\left(TPR\right)=\frac{TP}{TP+FN}\;$$

(5) $$Precision=\frac{TP}{TP+FP}\;$$

(6) $$Dice\;Score\;\left(DSC\right)=\frac{2\times TP}{2\times TP+FN+FP}\;$$

(7) $$Intersection\text{-}over\text{-}Union\;\left(IoU\right)=\frac{Area\;of\;Overlap}{Area\;of\;Union}=\frac{TP}{TP+FP+FN}\;$$

$T_{P}$ represents the true positive, $T_{N}$ is true negative, whereas $F_{P}\;and\;F_{N}$ are the false positive and false negative, respectively.

### 3.2. Performance Evaluation Analysis

**Figure 4:**
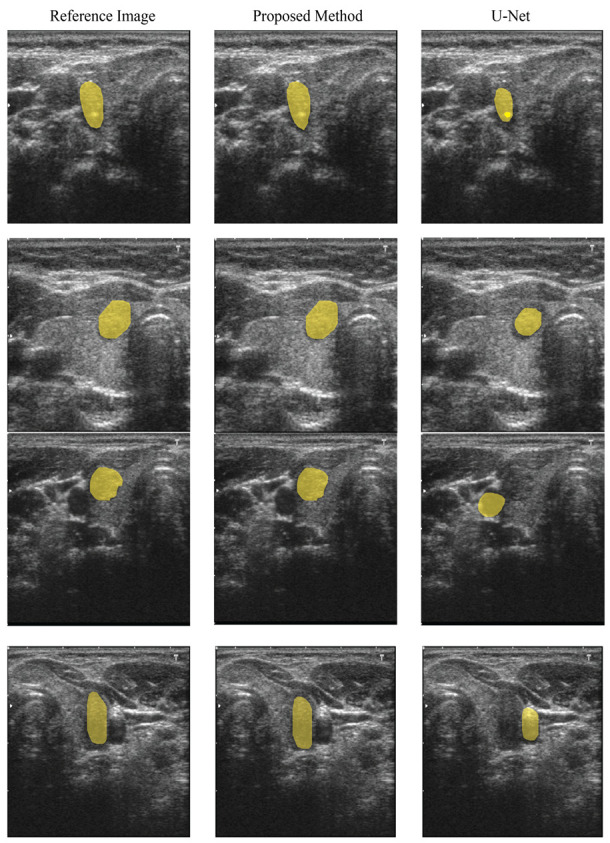
Segmentation output results of the proposed and benchmark methods.

This research aims to create a deep learning framework that can recognize thyroid nodules in ultrasound images effectively. Despite the considerable variances in the ultrasound data, the proposed model outperformed U-Net quantitatively and produced highly accurate detection results. The testing results revealed that the proposed model achieved an overall accuracy of 99%, dice score of 97.5%, sensitivity of 98%, IoU of 97.1%, and precision of 97%. In comparison, the benchmark U-Net approach obtained an accuracy of 96%, a precision of 96%, sensitivity of 95.2%, dice score of 95.4%, and IoU of 95.3%. Table 4 shows the comparison of both methods and their respective parameters. The experimental findings show that the proposed method is very accurate, efficient, and reliable. Figure 4 shows the qualitative segmentation results of the proposed method and the benchmark method (U-Net), demonstrating that the proposed method can segment thyroid nodules following the reference standard. In contrast, the state-of-the-art benchmark method (U-Net) cannot detect in some cases.

### 3.3. System Description and Time Analysis

The training phase was completed on the hardware unit with a Core i7-9750H@2.6 GHz processor with 16 GB DDR4 RAM. The graphics card used was NVIDIA RTX 2070. Furthermore, our suggested method exceeds the benchmark method in terms of computing efficiency. According to the processing time, the suggested VGG-16 and benchmark algorithms consume 0.2 and 0.47 s for inference time per test image, respectively. Furthermore, existing research shows that the processing time of 47 s for the test image is insufficient for real-time findings.

### 3.4. Performance Comparison with Other State-of-the-Art Methods

Several researchers have addressed the thyroid nodule problem in their studies and proposed the best possible solution. Table 5 summarizes the performance of previous researches in comparison to our proposed technique. The results in this table show that our proposed study outperforms earlier studies in detecting thyroid nodules using ultrasound imaging.

## 4. Conclusions

Ultrasonic accurate segmentation of the thyroid nodule area is an indispensable prerequisite for the diagnosis of thyroid cancer. For this purpose, we developed a deep learning model that uses the VGG-16 framework as the backbone, which is extensively used in medicine for the automatic detection and segmentation of thyroid nodule images. We evaluated our method on a TDID challenging thyroid dataset having high noise, blurry boundaries, and no calipers. The experimental results showed that the proposed method outperformed the state-of-the-art U-Net model. The proposed segmentation network segmented the thyroid nodule accurately with an accuracy of 99% and provided more precise predictions. Although artificial intelligence will not replace physicians in the near years, clinical specialists can study the principles of AI innovation and how AI-based structures can assist them in giving more benefits to their patients at work. In general practice, our deep learning model could help endocrinologists by providing a second opinion throughout the diagnosing process.

## Figures and Tables

**Table 1:** Distribution of dataset for Testing and Training.

Diagnosis Class	Training	Testing	Total
TIRADS-1	84	44	128
TIRADS-2	61	32	93
TIRADS-3	47	19	66
TIRADS-4	43	11	54
TIRADS-5	45	14	59
Total	280	120	400

**Table 2:** Network Parameters Detail.

Model	Learning Rate	Drop Rate	Weight Decay
Proposed Method	1 × 10^−9^	0.5	0.0005
U-Net	0.0001	0.2	0.0002

**Table 3:** Architecture and configuration of the proposed network.

Layer	Feature (Train)	Feature (Inference)	Kernel Size	Stride
Input Image	512 × 512 × 1	512 × 512 × 1	-	-
Padding	712 × 712 × 1	712 × 712 × 1	-	-
Convolutional 1				
Conv-1 + Relu-1	710 × 710 × 64	710 × 710 × 64	3 × 3	1
Conv-2 + Relu-2	710 × 710 × 64	710 × 710 × 64	3 × 3	1
Pool-1	355 × 355 × 64	355 × 355 × 64	2 × 2	2
Convolutional 2				
Conv-1 + Relu-1	355 × 355 × 128	355 × 355 × 128	3 × 3	1
Conv-2 + Relu-2	355 × 355 × 128	355 × 355 × 128	3 × 3	1
Pool-2	178 × 178 × 128	178 × 178 × 128	2 × 2	2
Convolutional 3				
Conv-1 + Relu-1	178 × 178 × 256	178 × 178 × 256	3 × 3	1
Conv-2 + Relu-2	178 × 178 × 256	178 × 178 × 256	3 × 3	1
Conv-3 + Relu-3	178 × 178 × 256	178 × 178 × 256	3 × 3	1
Pool-3	89 × 89 × 256	89 × 89 × 256	2 × 2	2
Convolutional 4				
Conv-1 + Relu-1	89 × 89 × 512	89 × 89 × 512	3 × 3	1
Conv-2 + Relu-2	89 × 89 × 512	89 × 89 × 512	3 × 3	1
Conv-3 + Relu-3	89 × 89 × 512	89 × 89 × 512	3 × 3	1
Pool-4	45 × 45 × 512	45 × 45 × 512	2 × 2	2
Convolutional 5				
Conv-1 + Relu-1	45 × 45 × 512	45 × 45 × 512	3 × 3	1
Conv-2 + Relu-2	45 × 45 × 512	45 × 45 × 512	3 × 3	1
Conv-3 + Relu-3	45 × 45 × 512	45 × 45 × 512	3 × 3	1
Pool-5	23 × 23 × 512	23 × 23 × 512	2 × 2	2
Conv-6 + Relu-6 + Drop6	17 × 17 × 4096	17 × 17 × 4096	7 × 7	1
Conv-7 + Relu-7 + Drop7	17 × 17 × 4096	17 × 17 × 4096	1 × 1	1
Conv-8	17 × 17 × 1	17 × 17 × 1	1 × 1	1
Deconv-9	576 × 576 × 1	576 × 576 × 1	64 × 64	32
Cropping	512 × 512 × 1	512 × 512 × 1	-	-
Output	512 × 512 × 1	512 × 512 × 1	-	-

**Table 4:** Evaluation Analysis of the Proposed and benchmark method.

Parameters	Proposed Method	U-Net
Accuracy	99	96
Precision	97	96
Sensitivity	98	95.2
DSC	97.5	95.4
IoU	97.1	95.3

**Table 5:** Comparison of the proposed method with other existing studies.

Authors	Method	Dataset	Accuracy (%)
Huitong et al. [25]	SGUNET	TDID	93.6
Wu et al. [26]	U-Net (backbone)	Private Dataset	93.19
Haji et al. [27]	SSHOS	TDID	96
Abdolali et al. [18]	Mask R-CNN	Private Dataset	84
Liu et al. [15]	ResNet-50 (backbone)	Private Dataset	97.1
Nguyen et al. [8]	ResNet + InceptionNet	TDID	92.05
Proposed Method	VGG-16 (backbone)	TDID	99

## Data Availability

Not applicable.
